# P-2165. Comparison of Incidence Reporting and Outcomes of Infections in Chimeric Antigen Receptor T (CART) Cell Therapy and Bispecific Antibodies (BsAb) Therapy for Multiple Myeloma: A Retrospective Pharmacovigilance Study

**DOI:** 10.1093/ofid/ofaf695.2328

**Published:** 2026-01-11

**Authors:** Varshini Thiruvadi, Arankesh Mahadevan, Saba Asif, Denise Francisco

**Affiliations:** University of Illinois College of Medicine, Peoria, Peoria, IL; University of Utah, Salt lake city, Utah, US, Salt lake city, Utah; Trinity Health Oakland/ Wayne State University, Pontiac, Michigan; University of Illinois College of Medicine, Peoria, Peoria, IL

## Abstract

**Background:**

Chimeric Antigen Receptor (CAR) T cell and Bispecific Antibody (BsAb) therapies have revolutionized the management of multiple myeloma (MM). However, they are associated with an increased risk of infections, particularly due to lymphopenia, neutropenia, Immune-Effector Cell-Associated Neurotoxicity Syndrome (ICANS), Cytokine Release syndrome (CRS), and Immune-effector cell-associated hemophagocytic lymphohistiocytosis (IECHLH).

**Table 1:**
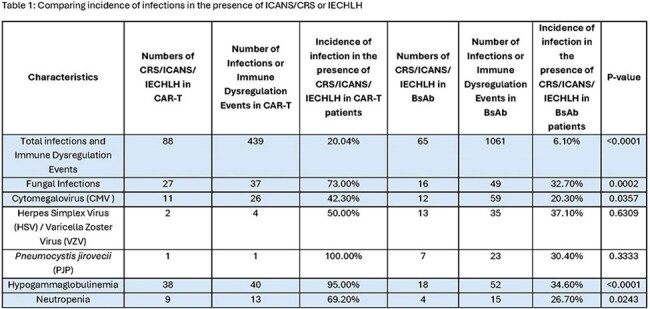
Comparing incidence of infections in the presence of ICANS CRS or IECHLH

**Figure 1:**
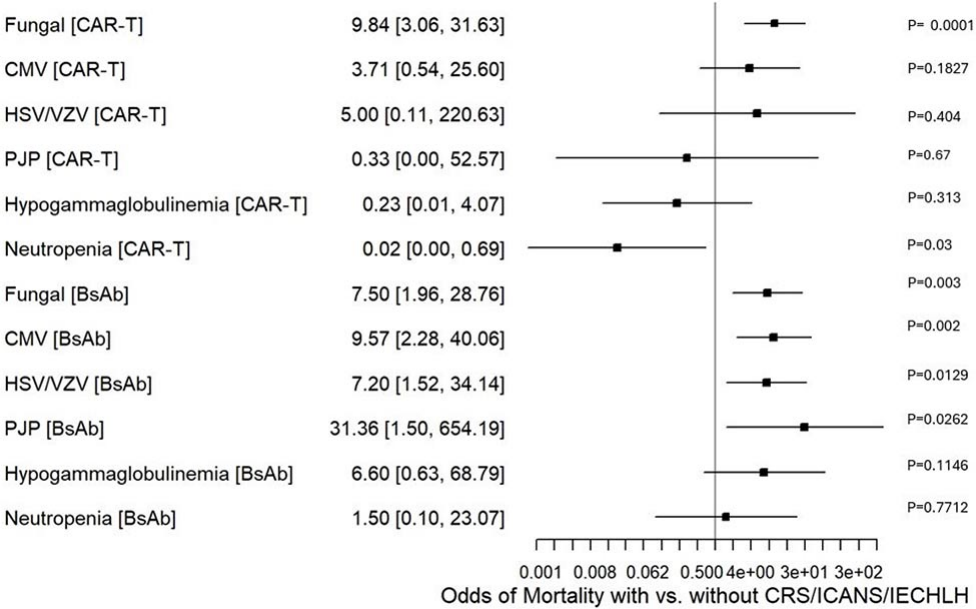
Comparing mortality outcomes from infections and immune dysregulation in the presence of CRS/ICANS/IECHLH in CART versus BsAb group represented on Forest Plot

**Methods:**

The FDA Adverse Event Reporting System (FAERS) database was accessed in April 2025 to conduct the study. Therapies analyzed were CAR-T (Ciltacabtagene, Idecabtagene) and BsAb constructs (Teclistamab, Talquetamab, Elranatanab). The number of patients, cases of various infections, and immune dysfunction events were collected for each group. The association of infections with CRS, ICANS, or IECHLH was analyzed. Infection rates were calculated as the proportion of reported events per total event following therapy. Categorical comparisons between therapies were conducted using chi-square or Fisher’s exact tests. Odds ratios with 95% confidence intervals were calculated for outcomes of infections in the presence of CRS, ICANS, or IECHLH for CAR-T versus BsAb comparisons. Statistical significance was set at a two-sided p-value < 0.05.

**Results:**

The total incidence of infections and immune dysregulation in the CAR-T versus BsAb group was 14.40% and 32.20%, statistically significant (p< 0.01). Hypogammaglobulinemia had the highest incidence in the CAR-T group (1.31%), followed by fungal infections (1.20%), while cytomegalovirus infections (1.79%) were the highest in BsAb, followed by hypogammaglobulinemia (1.58%). Infections and immune dysregulation in the presence of CRS, ICANS, or IECHLH were higher in CAR-T than in BsAb (20.0% vs 6.10%; p< 0.01) (Table 1). The odds ratio comparing differences in outcomes in patients with infections in the presence of CRS, ICANS, or IECHLH is illustrated in the forest plot (Figure 1).

**Conclusion:**

CAR-T and BsAb therapies are game-changers in treating MM but have drawbacks. Infections are the leading cause of non-relapse mortality in this group, further complicated by immune syndromes. This study underscores the importance of consensus on

anti-infective prophylaxis in this patient population.

**Disclosures:**

All Authors: No reported disclosures

